# Reconfigurable and nonvolatile photo-pyroelectricity in a ceramic-like biaxial molecular ferroelectric via polarization engineering

**DOI:** 10.1126/sciadv.aec5864

**Published:** 2026-05-29

**Authors:** Liwei Tang, Jialu Chen, Yu Ma, Yi Liu, Jingtian Zhang, Junhua Luo, Zhihua Sun

**Affiliations:** ^1^ State Key Laboratory of Functional Crystals and Devices, Fujian Institute of Research on the Structure of Matter, Chinese Academy of Sciences, Fuzhou, Fujian 350002, China.; ^2^ University of Chinese Academy of Sciences, Beijing 100049, China.; ^3^ Fujian Science & Technology Innovation Laboratory for Optoelectronic Information of China, Fuzhou, Fujian 350108, China.

## Abstract

Photo-pyroelectric effect provides a promising avenue to enhance optoelectronic device performance, in which reconfigurable and nonvolatile characteristics enable newly conceptual photoelectric memories. However, achieving reconfigurable and nonvolatile photo-pyroelectricity in molecule-based systems remains highly challenging because of the deterioration of pyroelectricity and fatigue resistance. Herein, we modulate the photo-pyroelectricity of a ceramic-like molecular ferroelectric, (*N*-methylcyclohexylammonium)_2_PbCl_4_ (**1**), showing biaxial ferroelectricity (Aizu notation: 4/*mmmFmm*2). The photo-pyroelectric current is enhanced from approximately 0.5 to 75 picoampere through external polarization engineering, corresponding to an improvement of nearly two orders of magnitude. Strikingly, positive-to-negative switching of the current direction verifies reconfigurable and nonvolatile merits. Under electric fields, its photo-pyroelectric responses can be deliberately paused and reactivated, giving newly conceptual optically readable memory “1/0” states. These reconfigured multistates exhibit exceptional nonvolatile retention exceeding 63 hours with anti-fatigue performance. As an innovative work, our study sheds light on exploration of ferroelectrics in memory storage and neuromorphic vision applications.

## Teaser

Photo-pyroelectric effect in a biaxial ferroelectric enables reconfigurable and nonvolatile multistate photoelectric memories.

## INTRODUCTION

Photo-pyroelectric effect, which couples pyroelectricity and light excitation to achieve photo-to-electric conversion, has emerged as a principle for developing the next-generation optoelectronic technologies (*1*–*3*). In addition to traditional energy harvesting and photodetection, programmable photosensing is one of the most crucial building units for the development of memory storage, computation, and neuromorphic vision systems (*4*–*8*). However, the current strategy using the compositional doping or heterostructure engineering usually leads to compromised pyroelectric coefficient or limited tunability (*9*, *10*), thus posing substantial challenges for the realization of reconfigurable and nonvolatile activities. As the most notable subclass of the pyroelectric family, ferroelectrics enable the couplings between spontaneous polarization (*P*_s_) and light, which is fundamental for their multifunctional photoelectronic properties, including optical nonlinearity, electro-optics, and photo-pyroelectric effect (*11*–*14*). In this scenario, it is proposed that the magnitude and direction of photo-pyroelectric currents will be controlled by electrically regulating ferroelectric *P*_s_ states, and their inherent polarization endows nonvolatile photo-pyroelectric activities. Therefore, the ferroelectric polarization engineering with inherent nonvolatility and directional controllability provides a feasible avenue to precisely modulate the photo-pyroelectric effect toward photoelectric memories and neuromorphic vision applications.

In recent years, molecular ferroelectrics have been widely explored as a class of functional materials with flexible structures and tunable polarization behaviors (*15*, *16*), among which molecule-based ferroelectrics of two-dimensional (2D) hybrid perovskites have recently received extensive interest, owing to their unique merits of structural compatibility, molecular tunability, and low-temperature thin film integration (*17*–*20*). Nevertheless, the performances of this 2D ferroelectric family are still far below the practical application level, despite the ongoing endeavors. The main obstacle of single-crystal application is how to break through the limitation caused by their inherent anisotropy and directional ferroelectricity. For example, the organic uniaxial ferroelectric of croconic acid single crystal has a large *P*_s_ comparable to that of BaTiO_3_ (*P*_s_ ~ 20 μC/cm^2^), while the remanent polarization (*P*_r_) value of its thin films sharply decreases to ~0.4 μC/cm^2^ (*21*, *22*). This similar polarization fatigue has also been found in LiNbO_3_, of which the application form is restricted to bulk crystal (*23*). Such inherent anisotropy of molecular uniaxial ferroelectric crystals also restricts the potentials of photo-pyroelectricity for device application. A newly emerging alternative is molecule-based multiaxial ferroelectrics, which have large *P*_s_ and giant piezoelectric coefficients, such as tetramethylammonium tetrachloroferrate(III), tetramethylammonium bromotrichloroferrate(III), and (pyrrolidinium)Ba(ClO_4_)_3_ (*24*, *25*). In contrast to uniaxial ferroelectrics, the multiaxial counterparts with multiple equivalent polarization directions enable the dynamic domain reorientation under external fields (e.g., polarization engineering) (*26*–*28*). This is especially advantageous in polycrystalline ceramic–like form, of which the polarization control is almost independent of grain orientation (*29*, *30*), thus allowing the precise modulation of photo-pyroelectric effect. Despite recent studies on photoactive 2D hybrid perovskite ferroelectrics, it remains a huge challenge to realize the reconfigurable and nonvolatile photo-pyroelectricity in these molecule-based systems.

Here, we have demonstrated the realization of the reconfigurable and nonvolatile photo-pyroelectric activities in a 2D biaxial ferroelectric (*N*-methylcyclohexylammonium)_2_PbCl_4_ (**1**), by virtue of polarization engineering to its ceramic-like polycrystalline wafers. The photo-pyroelectric current is enhanced by two orders of magnitude through controlling the polarization engineering. Notably, its multiaxial ferroelectric nature and independent domain orientation facilitate the positive-to-negative switching of current directions, confirming the reconfigurable and nonvolatile merits of **1**. Moreover, the photo-pyroelectric response can be altered at various degrees by applying different external electric fields, leading to the multistate memory behaviors (“1/0” states). These reconfigured multistates further give rise to an exceptional nonvolatility with the retention time beyond 63 hours, which suggests its potential in nonvolatile and writable photoelectric memories. The study on reconfigurable photo-pyroelectric orders of ceramic-like molecular ferroelectric provides an opportunity to exploit promising pathway for applications in memory storage and neuromorphic visual systems.

## RESULTS

### Structural analysis

**Fig. 1.**
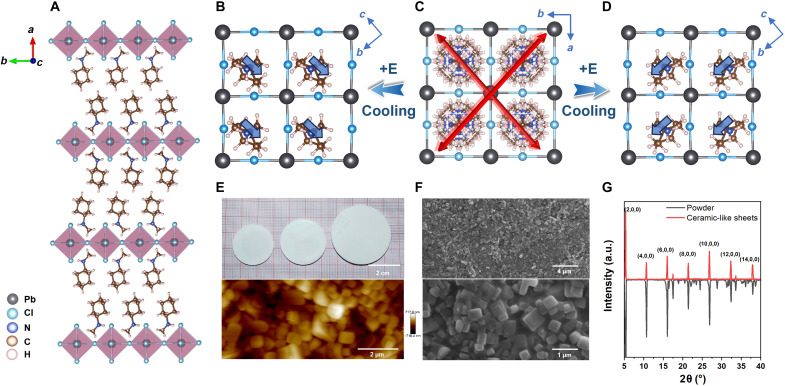
Structural analysis. (**A**) Structure packing diagram of **1** at FEP. (**B**) The *P*_s_ direction along the *c* axis at FEP. (**C**) Four equivalent [110] directions at PEP during symmetry breaking of 4/*mmmFmm*2. (**D**) Variation of the *P*_s_ direction under an applied electric poling. (**E**) Optical image of ceramic-like sheets of **1** and AFM image. (**F**) SEM images. (**G**) XRD patterns of the ceramic-like sheets and powder data. a.u., arbitrary units.

Differential scanning calorimetry measurements confirm that **1** undergoes two successive phase transitions at 361/357 K (*T*_c1_) and 373/370 K (*T*_c2_) in the heating/cooling runs (fig. S1). Variable-temperature structure analysis reveals that **1** adopts a typical 2D monolayered Ruddlesden-Popper perovskite motif (Fig. 1A), and its paraelectric phase (PEP) crystallizes in space group of *I*4/*mmm* at high temperature. Upon cooling, it undergoes two phase transitions to ferroelectric phase (FEP) with the polar space group of *Pmn*2_1_ (*31*). This transition corresponds to symmetry breaking from 4/*mmm* to *mm*2, as characterized by an Aizu notation 4/*mmmFmm*2. The number of symmetric elements decreases from 16 (*E*, 2*C*_4_, *C*_2_, 2*C*_2_′, 2*C*_2_″, *i*, 2*S*_4_, σ_h_, 2σ_v_, 2σ_d_) to 4 (*E*, *C*_2_, σ_v_, σ_v_), coinciding well with the Landau phase transition theory (*32*). In the FEP, **1** exhibits polarization constrained along the crystallographic *c* axis (Fig. 1B). In contrast, in the PEP, it crystallizes in the symmetric space group with two symmetry-equivalent axes, leading to four possible equivalent orientations: [110], [$\bar{1}$10], [1$\bar{1}$0], and [$\bar{1}\bar{1}$0] (Fig. 1C). During the symmetry-breaking phase transition upon cooling, one of those equivalent axes becomes the polarization axis of the FEP. Without an external electric field, this selection occurs randomly across the crystal. However, under an applied electric field, the polarization axis can be directed to align its polar axis parallel to the applied electric field in a controllable manner (Fig. 1, B and D). That is, **1** exhibits four equivalent orientations and two ferroelectric axes, indicating its multiaxial nature, which is crucial for polarization switching and domain engineering. In practical applications of ferroelectric materials, thin films and polycrystalline ceramics are commonly used forms (*12*, *33*), so a greater number of equivalent polarization directions are preferred to allow the *P*_s_ to orientate along the external electric field. Ceramic-like sheets of different sizes were easily prepared by application of a pressure of ∼5 MPa at room temperature (Fig. 1E). Figure 1G illustrates the x-ray diffraction (XRD) diffraction data of the ceramic-like sheet, and most of the diffraction peaks are represented by the (*h*00) index, indicating that the ceramic-like sheet is highly oriented. Surface characterization using atomic force microscopy (AFM) and scanning electron microscopy (SEM) demonstrates a uniform distribution of grains with clearly defined grain boundaries (Fig. 1, E and F), which is essential for achieving consistent electrical and thermal properties in the polycrystalline forms.

### Multiaxial ferroelectric properties

**Fig. 2.**
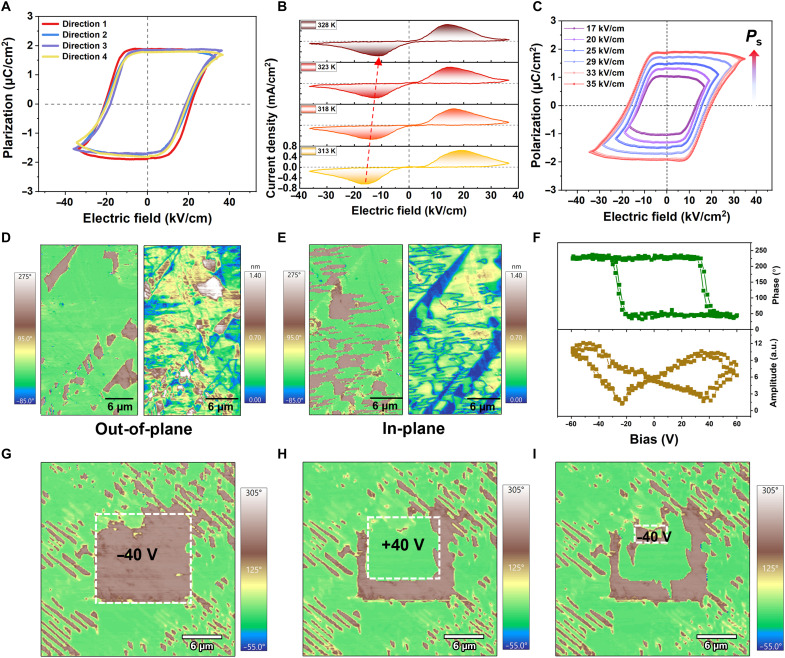
Ferroelectric properties. (**A**) *P-E* hysteresis loops at different directions. (**B**) *J-E* ferroelectric hysteresis loops at different temperatures. (**C**) *P-E* hysteresis loops measured at different electric fields. (**D**) Vertical PFM (left) phase and (right) amplitude. (**E**) Lateral PFM (left) phase and (right) amplitude on the thin film of **1**. (**F**) Local PFM hysteresis loops obtained by plotting the phase and amplitude signals as functions of the tip voltage for a selected point. (**G** to **I**) Vertical PFM phase on the thin film of **1** were obtained by performing continuous polarization switching within the central square region. The topographic images and PFM amplitude images on the thin film of **1** during ferroelectric domain switching are all presented in fig. S6.

Multiaxial ferroelectrics enable reorientation of the polar axis of the crystal and exhibit effective polarization switching even in polycrystalline form. This stands in marked contrast to conventional uniaxial molecular ferroelectrics: The polarization switching of 180° flipping is achievable only in well-oriented single crystals with geometries conducive to the application of an external electric field (*22*). Well-defined electric field (*P-E*) ferroelectric hysteresis loops can be measured along any direction in the polycrystalline forms of **1** (Fig. 2A and fig. S2). This isotropic ferroelectric behavior in the polycrystalline form is highly advantageous for practical applications. Temperature-dependent measurements of the *P-E* hysteresis loops and current-electric field (*J-E*) curves reveal that both the coercive field (*E*_c_) and *P*_s_ decrease with increasing temperature (Fig. 2B and fig. S3), consistent with the typical behavior of ferroelectric materials approaching the Curie temperature (*T*_c_). When the amplitude of the applied electric fields marginally surpasses the *E*_c_, the hysteresis loop with a relatively small *P*_s_ (~0.8 μC/cm^2^) is obtained, which is attributed to the random orientation of polarization axes in each crystalline grain. As the strength of the electric field increases, the magnitude of *P*_s_ gradually increases and eventually approaches a value close to that of single crystal (Fig. 2C) (*31*), highlighting the unique advantages of the polycrystalline form for performance modulation and device integration. In contrast, the single crystal exhibits no notable change in *P*_s_ with increasing electric field (fig. S4). These results underscore the potential of the polycrystalline form of **1** for applications requiring flexible polarization control.

Piezoresponse force microscopy (PFM) is a powerful tool that enables the direct visualization of ferroelectric domain structures at the micro- and nanoscale. Figure 2 (D and E) and fig. S3 record the original vertical and lateral PFM images of a region on the film of **1**. The phase images (Fig. 2 D, left, and E, left) reveal two distinct types of domains with clear phase contrast in both out-of-plane and in-plane orientations, and the domain patterns differ between two orientations. In the corresponding amplitude images (Fig. 2, D, right, and E, right), the domain walls are distinctly visible and align well with the domain boundaries identified in the phase images. Moreover, the domain structures observed in both orientations do not correlate with the topographic features (fig. S5), confirming that the domain contrast originates from intrinsic ferroelectric domains rather than surface morphology. The difference between the out-of-plane and in-plane domain patterns indicates the presence of non-180° domains, which uncovers the coexistence of multiple polarization orientations of **1**. We also applied a direct current bias to induce polarization switching, followed by PFM measurements under alternating current voltages. This procedure yields the characteristic amplitude-bias butterfly curves and phase-bias hysteresis loops (Fig. 2F), confirming reversible polarization switching behavior of the domains. For more intuitive evidence, we performed a series of domain-switching experiments (Fig. 2, G to I, and fig. S6). In Fig. 2G and fig. S6, A and B, after applying the −40-V voltage to a central square region on the pristine domains, the subsequent PFM phase and amplitude images show that the central area largely switched to a brown-colored domain state. A subsequent application of the +40-V voltage to a smaller square within the previously switched region resulted in the emergence of a green domain in the phase image (Fig. 2H), with newly formed domain walls clearly observed in the corresponding amplitude image (fig. S6D), indicating successful domain switching. Last, reapplying a −40-V voltage to the central area restores the brown domain (Fig. 2I and fig. S6, E and F), demonstrating reversible ferroelectric switching under an external electric field. Owing to the multiaxial nature, the direction with small component can be switched to the one with large component, thus enhancing performance in the ceramic-like polycrystalline form. Such a phenomenon might account for the relatively high *P*_s_ value of the ceramic-like sheet of **1**, which approaches that of the single crystal counterpart.

### Reconfigurable photo-pyroelectricity

**Fig. 3.**
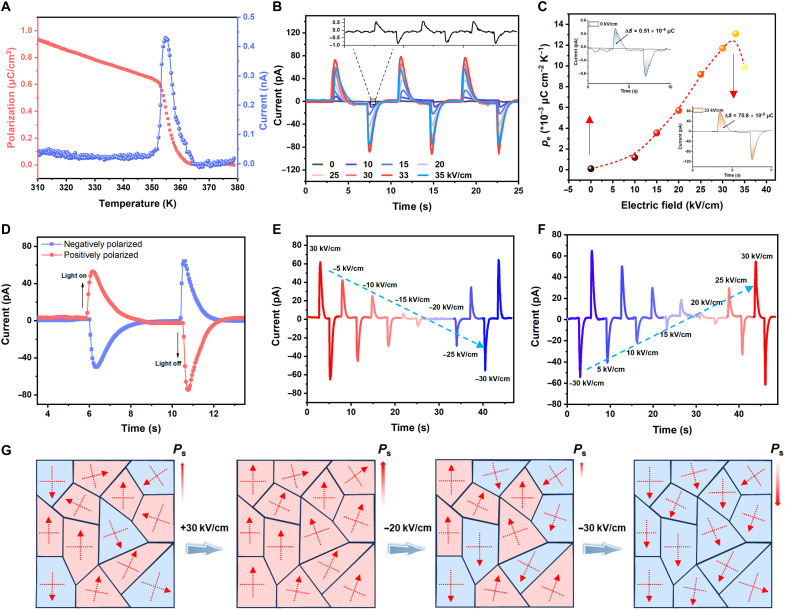
Reconfigurable photo-pyroelectricity. (**A**) Temperature-dependent *P*_s_ and pyroelectric currents. (**B**) Photo-pyroelectric currents measured at different electric field under 404-nm laser illumination (135 mW/cm^2^, at zero bias). (**C**) Pyroelectric coefficient (*p*_e_) at different electric fields, insert: on-off response cycle (0 and 33 kV/cm). (**D**) Photo-pyroelectric currents measured at 30 and −30 kV/cm. (**E** and **F**) Illuminated *I-t* curves measured after applying positive and negative pulses with different electric field, respectively. (**G**) Domain structures in polycrystals multiaxial ferroelectrics. Each microcrystal is enclosed by bold boundaries and consists of multiple ferroelectric domains with distinct polarization directions.

Ferroelectrics are a subclass of pyroelectric materials, and all ferroelectrics show pyroelectricity (*34*). As shown in Fig. 3A, the steady-state pyroelectric measurements of the polycrystalline form reveal a sharp current peak near the *T*_c_ of **1**, giving a pyroelectric coefficient (*p*_e_) of ~0.95 μC/cm^2^ at *T*_c_ (fig. S6). Next, we explored the photo-pyroelectric effect of the **1**-based polycrystalline forms under 0-V bias voltage condition (fig. S8). As demonstrated in the inset of Fig. 3B and figs. S9 and S10, under 404-nm illumination (135 mW/cm^2^), the current-time (*I-t*) and voltage-time (*V-t*) characteristics exhibit an obvious photo-pyroelectric effect, with positive pyroelectric current (*I*_pyro_) 0.5 pA and negative pyroelectric voltage (*V*_pyro_) −12 mV, slightly smaller than the single crystal sample (fig. S11). The rising and falling components of response time (τ_rise_/τ_fall_) deduced from one switching cycle are estimated as 200 and 224 ms, respectively (fig. S12). The emergence of these peaks originates from rapid surface temperature fluctuations induced by light illumination, which results in changes in the effective polarization and surface potential. To get the polarization-controlled photo-pyroelectric response, we measured the *I-t*/*V-t* curves after polarization with different applied electric fields. The *I*_pyro_ and *V*_pyro_ increase monotonically with the poling electric field strength up to 33 kV/cm (Fig. 3B and fig. S10). When the electric field exceeds a certain threshold, irreversible defects, such as domain wall pinning or microcracks, are introduced within the material, leading to a degradation of photo-pyroelectric response. Polarization engineering results in two orders increase in *I*_pyro_, *V*_pyro_, and *p*_e_ (Fig. 3, B and C, and figs. S8 and S13). Furthermore, the direction of the *I*_pyro_ and *V*_pyro_ can be reversibly switched by changing the electric field direction (Fig. 3D and fig. S14). The electric field–induced and reversible switching of ferroelectric polarization enables nonvolatile reconfiguration of both the magnitude and direction of the photo-pyroelectric response. The reconfigurable modulation of the photo-pyroelectric effect of **1** by ferroelectric polarization was further investigated using continuous electric poling. Before continuous poling, preset pulses of +30 or −30 kV/cm can be applied to set the polarization upward or downward, respectively. As shown in Fig. 3E, when the initial pulse is +30 kV/cm, the device obtains an *I*_pyro_ of 61 pA. As the negative voltage increases, the *I*_pyro_ gradually decreases from positive to zero and then turns negative. When the negative voltage increases to −30 kV/cm, the device has an *I*_pyro_ of −57 pA. This indicates a gradual change in the direction of polarization. In contrast, when the initial pulse is −30 kV/cm, as the positive pulse voltage increases, *I*_pyro_ gradually switches from −57 to 56 pA (Fig. 3F). The up-to-down and down-to-up polarization switching processes are rather symmetric. This observation further confirms the reversible control of polarization in modulating the reconfigurable photo-pyroelectric response.

The underlying mechanism can be elucidated through domain orientation switching (Fig. 3G). In the initial state, because of the random orientation of polarization axes within each grain, *P*_s_ exhibits a relatively low value, resulting in a small *I*_pyro_. As the amplitude of the applied electric field increases, the polarization axes in the polycrystalline material tend to align along the field direction, resulting in a gradual increase in *P*_s_. This realignment directly enhances the pyroelectric effect, as the *p*_e_ is intrinsically related to the temperature dependence of *P*_s_, expressed as

$$I_{\text{pyro}}=\frac{dQ}{dt}=p_{e}A\frac{dT}{dt}$$ (1)

$$p_{e}=\frac{dP_{s}}{\text{d}T}$$ (2)

where *Q* is pyroelectric charge, *A* is the surface area of the pyroelectric material, and *t* is time. Conversely, when an electric field of opposite polarity is applied, partial domain reversal occurs, leading to the coexistence of domains with antiparallel *P*_s_. This results in a net reduction—or even inversion—of the effective photo-pyroelectric response. Further, electric poling notably affects the piezoelectric properties of **1**. Before poling, the measured piezoelectric coefficient (*d*_33_) is 0.4 pC/N. Upon application of a 30 kV/cm electric field, *d*_33_ markedly increases to 3.4 pC/N (fig. S15), indicating that poling engineering not only enhances the photo-pyroelectric response but also enables the modulation of other polarization-related functionalities. This domain switching behavior highlights the potential of polycrystalline multiaxial ferroelectrics for reconfigurable photo-pyroelectric modulation.

### Photo-pyroelectric polymorphic memory

**Fig. 4.**
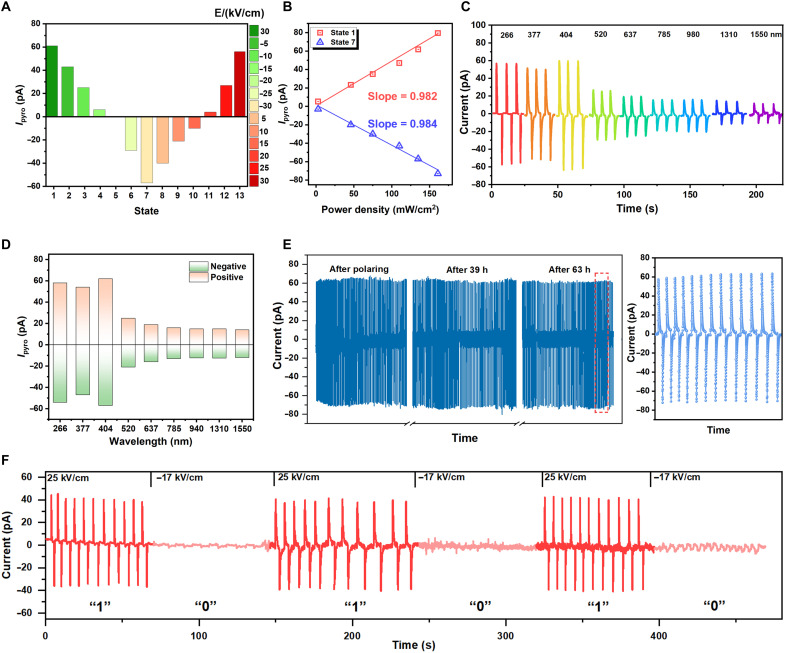
Photo-pyroelectric polymorphic memory. (**A**) Different nonvolatile photo-pyroelectric response states. (**B**) *I*_pyro_ as a function of optical power density with a highly linear relationship. (**C**) Photo-pyroelectric currents measured under 266- to 1550-nm laser illumination (~135 mW/cm^2^, at zero bias). (**D**) *I*_pyro_ measured under 266 to 1550 nm at 30 and −30 kV/cm. (**E**) Reproducible nature of the photo-pyroelectric currents generation (404 nm, 135 mW/cm^2^). (**F**) Photo-pyroelectric currents of the device were measured with 404-nm illumination (135 mW/cm^2^). Photo-pyroelectric currents were measured at 25 and −17 kV/cm. h, hours.

By applying the gradually increasing positive/negative polarization voltages, the reversible modulation of photo-pyroelectric response is achieved. We can therefore establish a one-to-one correlation between the photoresponsivity states and the polarization states (Fig. 4A), which promises to develop promising methods to implement multistate memory. Figure 4B and fig. S16 illustrate the measured relationships between photocurrent and light intensity for state 1 and state 7, all of which can be well represented by linear fits. Furthermore, the device displays broadband photoresponse (266 to 1550 nm), surpassing the limitation of conventional bandgap-constrained photodetectors (Fig. 4C and fig. S17). We characterized the photo-pyroelectric response under different wavelengths at different polarized electric fields (+30 to −30 kV/cm). The *I*_pyro_ at different wavelengths can be continuously tuned by the poling voltage (Fig. 4D), making it highly versatile for broadband applications. Critically, the modulated photocurrent state remains stable for more than 63 hours (Fig. 4E), which is a direct result of the inherent polarization nonvolatility of ferroelectric materials. To evaluate repeatability, photo-pyroelectric currents were measured under identical conditions on multiple devices under identical conditions, and they exhibit consistent anti-fatigue behavior and nonvolatile retention characteristics (fig. S18). Figure 4F describes the reproducible reversal of the *I*_pyro_ under short electrical pulses. The *I*_pyro_ is maintained at 45 pA under the applied electrical pulse of 25 kV/cm, which is defined as the “1” state. After applying a reverse poling pulse (−17 kV/cm), the *I*_pyro_ will drop to the level close to zero, which is defined as the “0” state. The experimental results are reproducibly observed across multiple devices (fig. S19). This dynamic modulation of *I*_pyro_ is expected to develop promising methods for achieving polymorphic memory. Our devices exhibit adjustable and controllable photo-pyroelectric response. This spectrally universal, nonvolatile, and multilevel photoresponse modulation not only gives an optically readable ferroelectric memory but also opens the way for neuromorphic sensors and adaptive optoelectronic systems based on the photo-pyroelectric effect.

## DISCUSSION

In conclusion, we have demonstrated a breakthrough in the reconfigurable and nonvolatile photo-pyroelectricity of biaxial ferroelectric ceramic–like polycrystalline sheets, achieved through precise polarization engineering. By controlling the alignment of ferroelectric domains, we realized two orders enhancement in photo-pyroelectric currents. Notably, the independent domain orientation switching of polycrystalline films allows the *I*_pyro_ to be continuously and reversibly modulated from positive to negative by poling engineering. This tunability is coupled with an exceptional nonvolatility, enabling the realization of nonvolatile multistate photo-pyroelectric memory. This study paves the way for further exploration of perovskite-based materials in nonvolatile, polymorphic memory applications.

## MATERIALS AND METHODS

### Synthesis

Pb(COOH)_2_·3H_2_O (10 mmol) and *N*-methylcyclohexylamine (20 mmol) were dissolved in 15 ml of HCl solution and stirred at 373 K for 30 min, yielding a clear solution. Plate-like colorless crystals of **1** were obtained using the temperature-cooling method at a rate of 1 K per day.

### Preparation of ceramic-like sheets

Crystals of **1** were first ground into powder and sieved. The obtained powder was then placed into a mold and pressed under a pressure of ~5 MPa for 1 min to obtain ceramic-like pellets.

### Preparation of thin films

Crystals of **1** (0.15 g) were dissolved in *N*,*N*′-dimethylformamide (0.66 g). Cleaned indium tin oxide–coated substrates were ultraviolet (UV)–ozone treated, then spin-coated (2000 rpm, 15 s) in a nitrogen glovebox, and annealed at 80°C for 10 min.

### Ferroelectric and PFM measurements

*P-E* hysteresis loops were measured using a Radiant Precision Premier II system with a Sawyer-Tower circuit. The electrical poling was performed at 25°C using the cyclic electric field with a pulse duration of 100 Hz. Domain structure and local switching behavior were characterized via PFM using a resonant-enhanced MFP-3D (Asylum Research) and Pt/Ir-coated silicon probes (EFM-50, Nanoworld).

### Characterization

AFM and SEM analyses were performed using a Bruker Dimension ICON and JEOL JSM6700-F, respectively. The XRD pattern of powder was obtained by the MiniFlex 600. Pyroelectric and photo-pyroelectric current were measured with a Keithley 6517B electrometer. The Chynoweth method was used to analyze the photo-pyroelectric current at room temperature. Light illumination was performed with laser diodes (L404P400M, L520P50, HL63142DG, L785P090, and L980P100A). UV and infrared light were provided by the handmade 266-nm laser (LDMQ-266-10) and laser diodes (PiL037X). The New Focus 1310 nm and the New Focus 1550-nm lasers (Velocity TLB-6730) were used for the infrared light illumination. Thermal imaging utilized the HIKVISION H21pro.
