# Supplementary material for: Reconfigurable and nonvolatile photo-pyroelectricity in a ceramic-like biaxial molecular ferroelectric via polarization engineering
Source: Sci Adv. 2026 May 29;12(22):eaec5864. doi: 10.1126/sciadv.aec5864 (PMC13220874; doi:10.1126/sciadv.aec5864)
Supplement: Supplementary file 1 — Figs. S1 to S19 Legend for data S1 [file sciadv.aec5864_sm.pdf]

Science Advances
AAAS

Supplementary Materials for

Reconfigurable and nonvolatile photo-pyroelectricity in a ceramic-like biaxial molecular ferroelectric via polarization engineering

Liwei Tang *et al.*

Corresponding author: Zhihua Sun, sunzhihua@fjirsm.ac.cn

Sci. Adv. **12**, eaec5864 (2026)
DOI: 10.1126/sciadv.aec5864

The PDF file includes:

Figs. S1 to S19
Legend for data S1

Other Supplementary Material for this manuscript includes the following:

Data S1

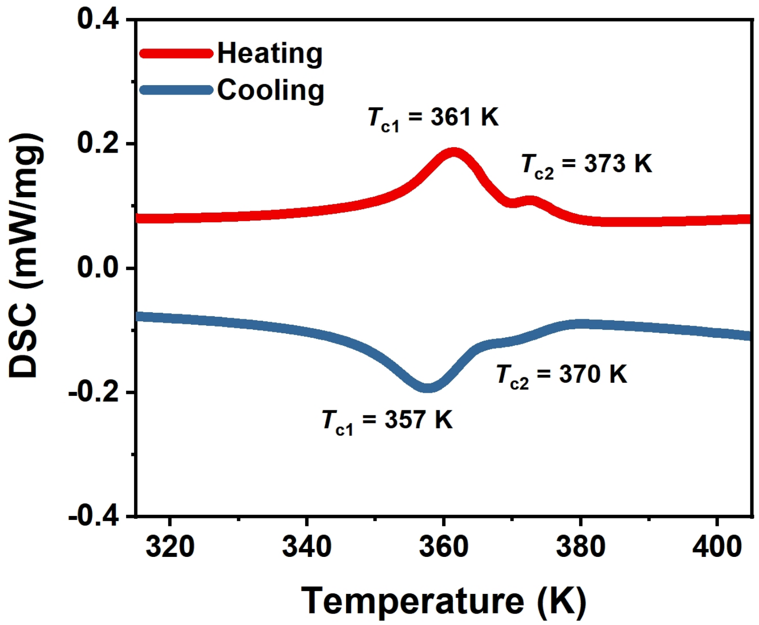

Fig. S1. DSC traces collected in the heating and cooling runs.

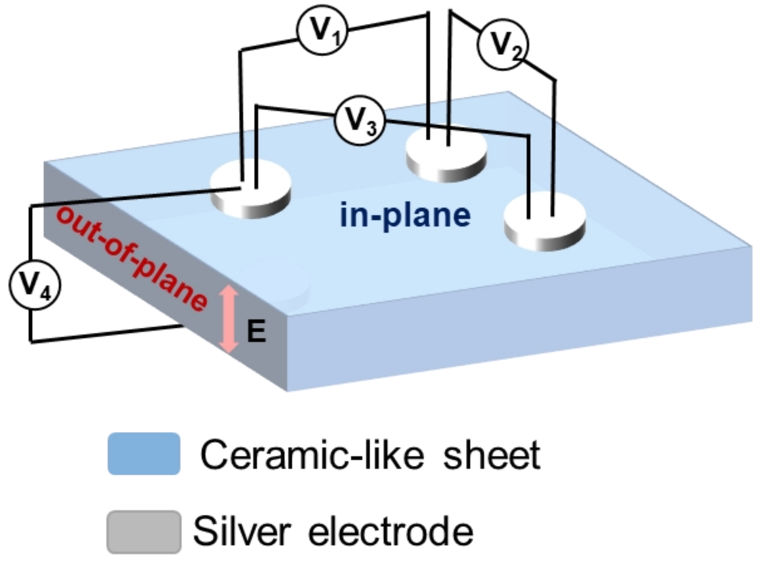

Fig. S2. Schematic diagram of ferroelectric measurement of ceramic-like sheets in different directions.

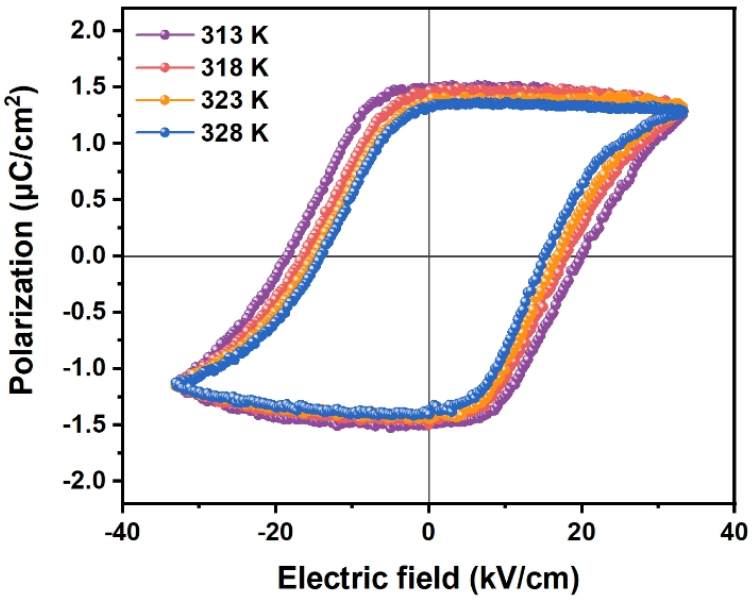

Fig. S3. Polarization versus electric field (*P-E*) ferroelectric hysteresis loops collected based on ceramic-like sheets.

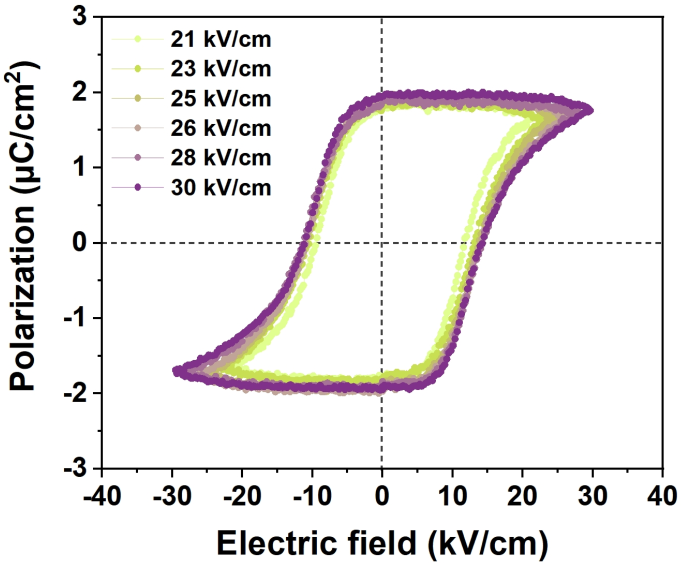

Figure S4: *P-E* hysteresis loops of single crystal measured at different electric fields.

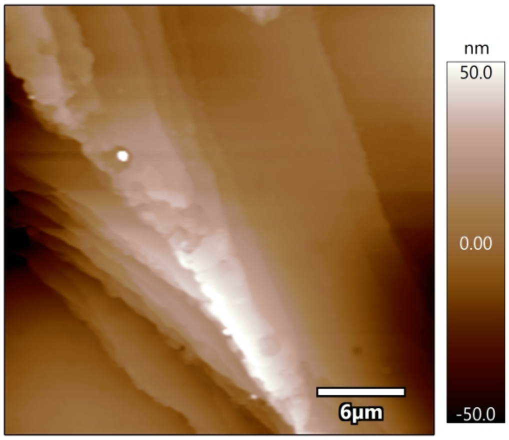

Fig. S5. Topographic images on the thin film of 1.

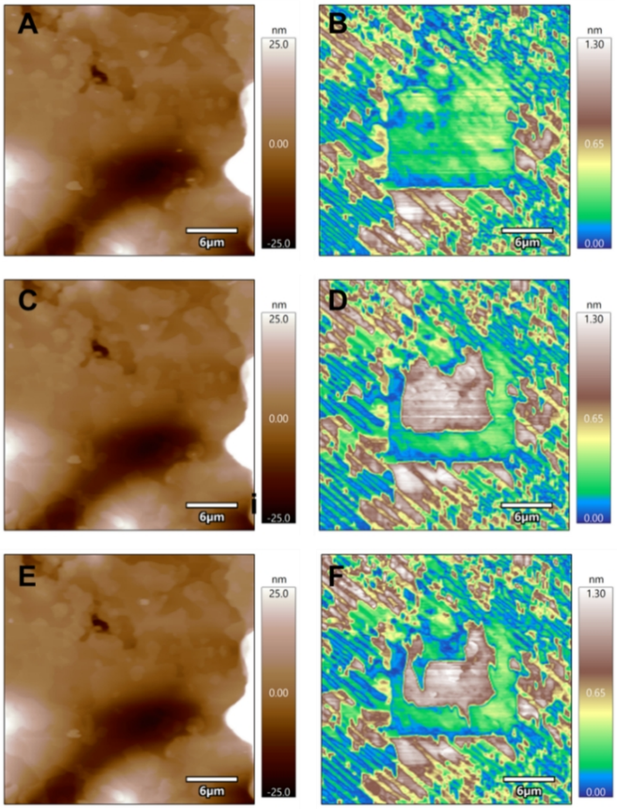

Fig. S6. Vertical PFM images on the thin film of 1 during ferroelectric domain switching. The topographic images (left) and PFM amplitude images (right) on the thin film of **1** were obtained under the following conditions: First, a bias of -40 V was applied to the middle square area (A, B). Subsequently, a +40 V bias was applied to the smaller square (C, D). Finally, a -40 V bias was applied to the smallest square (E, F).

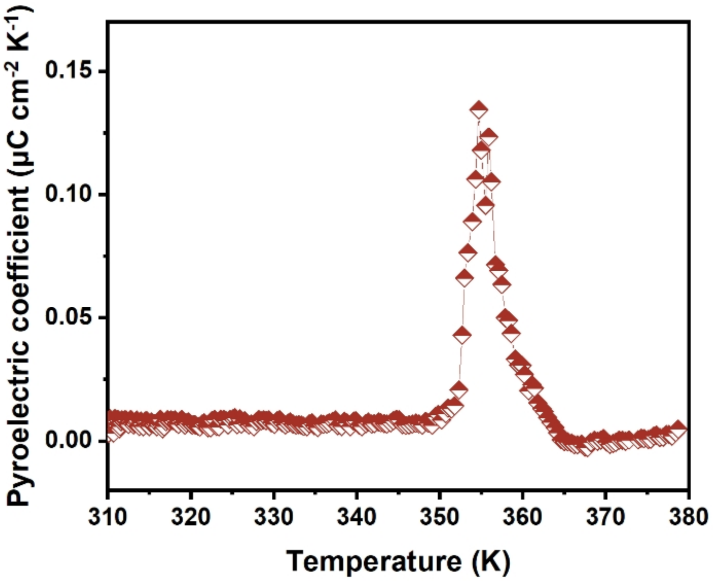

Fig. S7. Variable temperature of pyroelectric coefficient (p_e) trace.

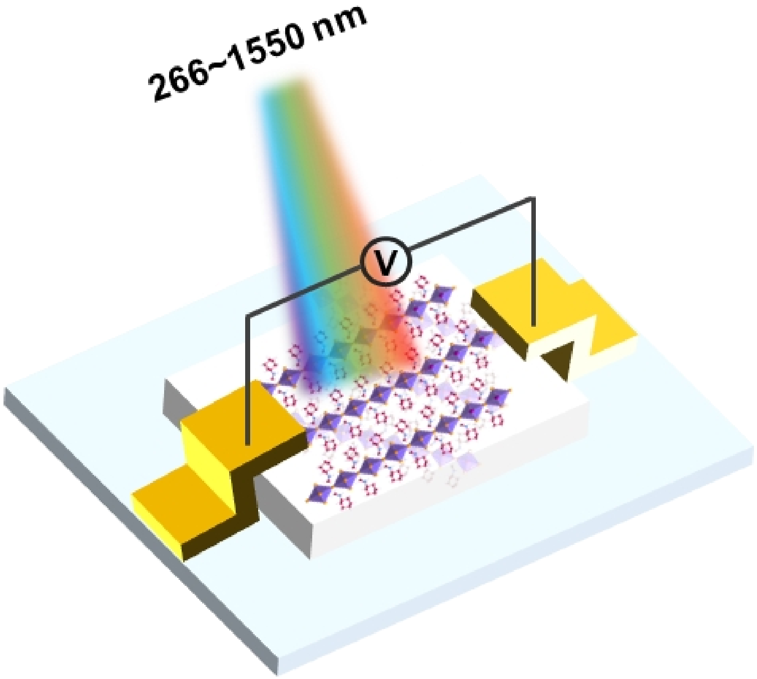

Fig. S8. Schematic diagram of polycrystalline form device.

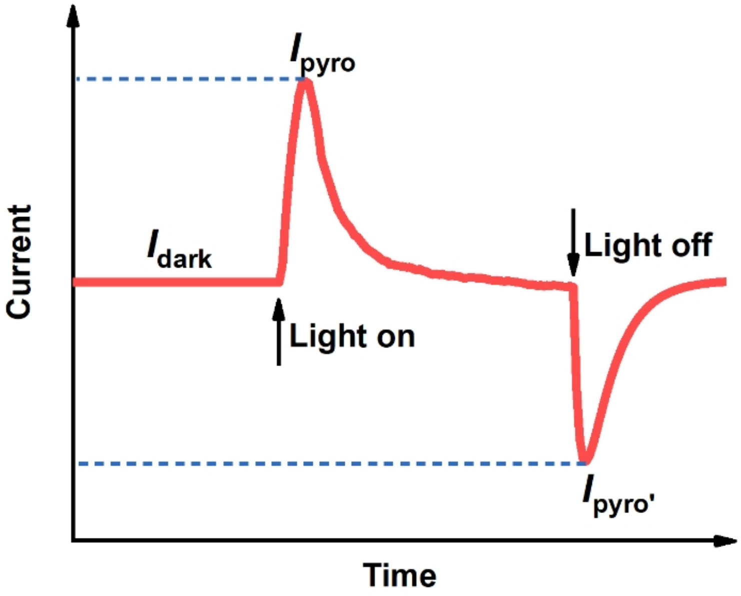

Fig. S9. A detailed view of a single cycle.

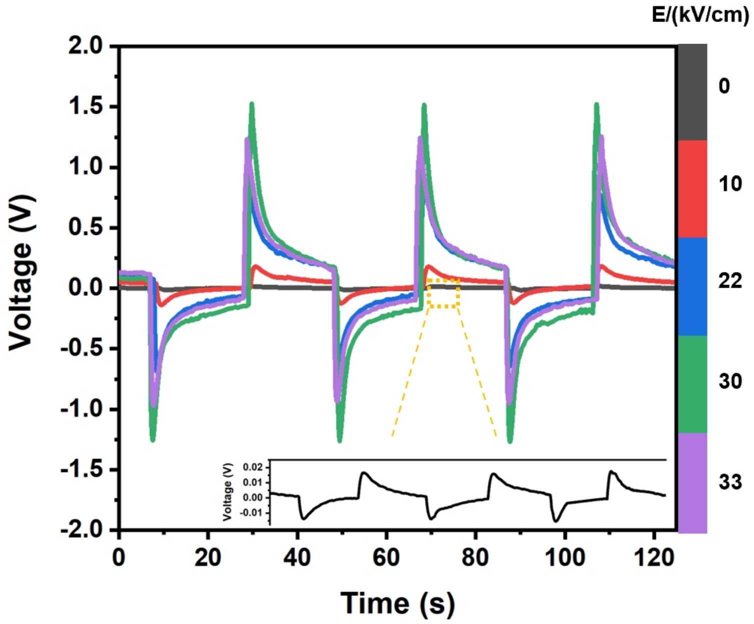

Fig. S10. Photo-pyroelectric voltages measured at different electric fields under 404 nm laser illumination (135 mW/cm^2, at zero bias).

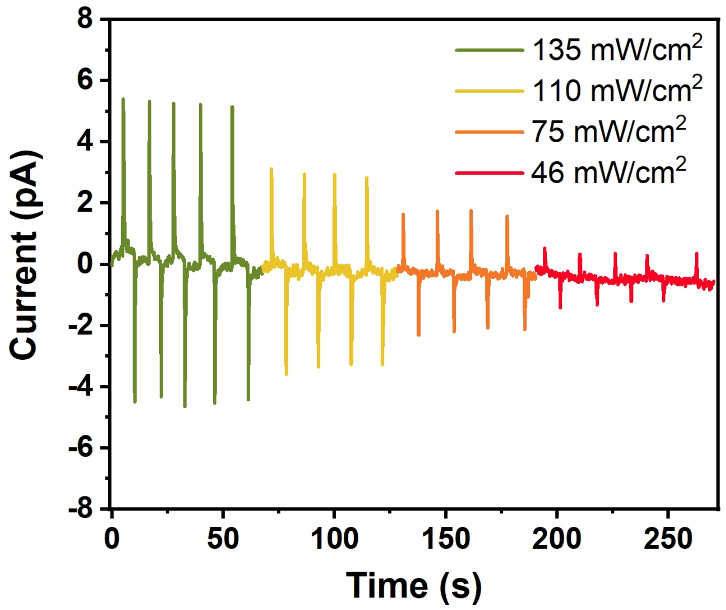

Fig. S11. Photo-pyroelectric currents of single crystal measured under 404 nm laser illumination with different light intensities (unpoled).

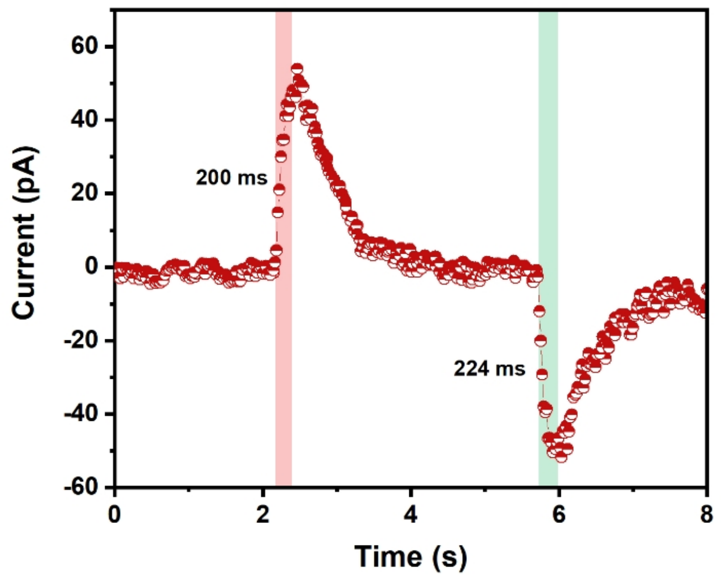

Fig. S12. The response time of 1 measured under 404 nm.

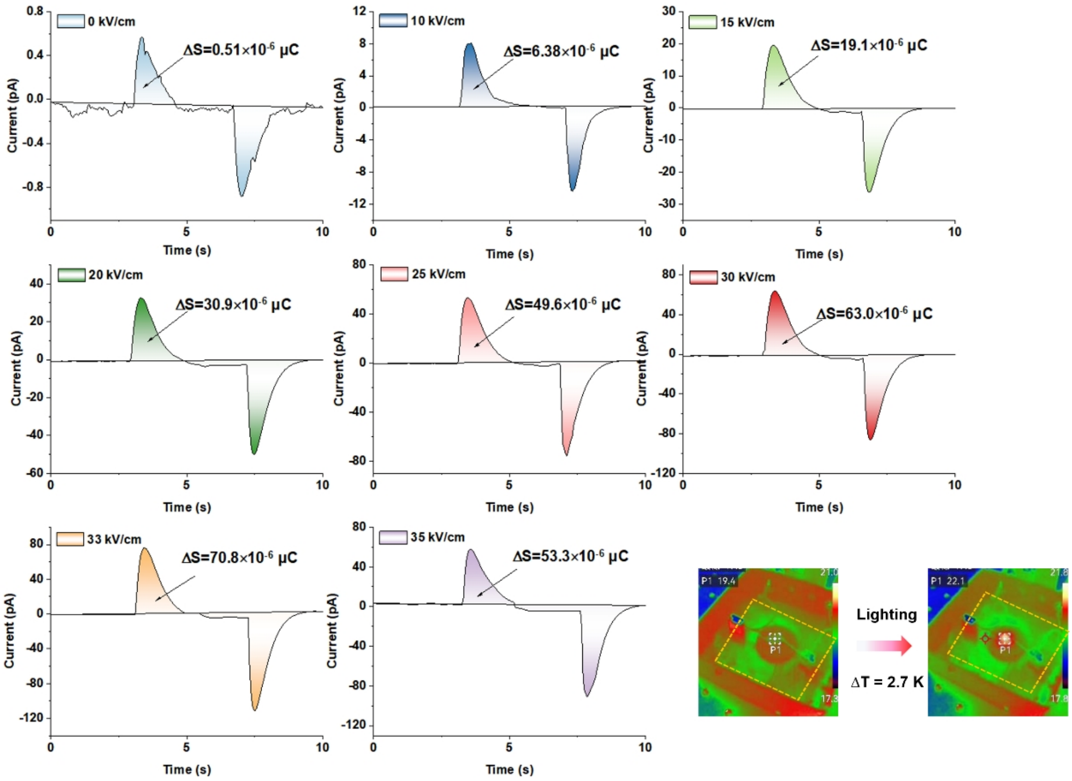

Fig. S13. On-off response cycle under different electric fields, and thermal imaging photos of device surface in the dark and irradiated condition.

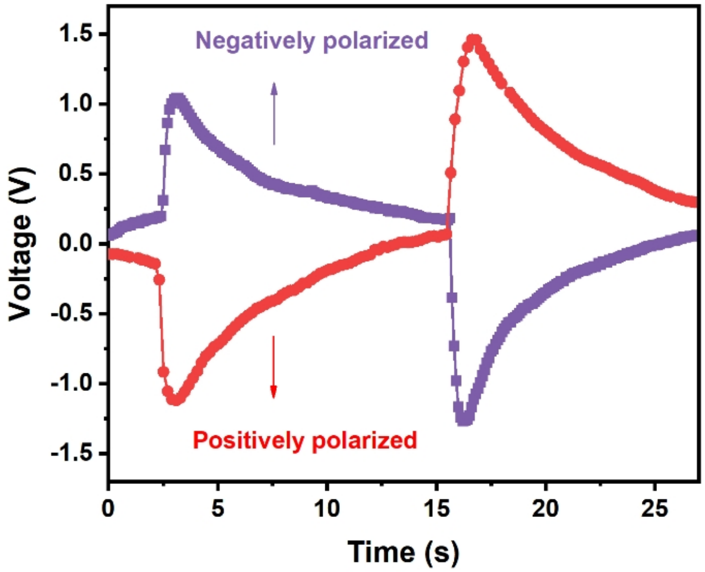

Fig. S14. Photo-pyroelectric voltages measured at 30 kV/cm and -30 kV/cm.

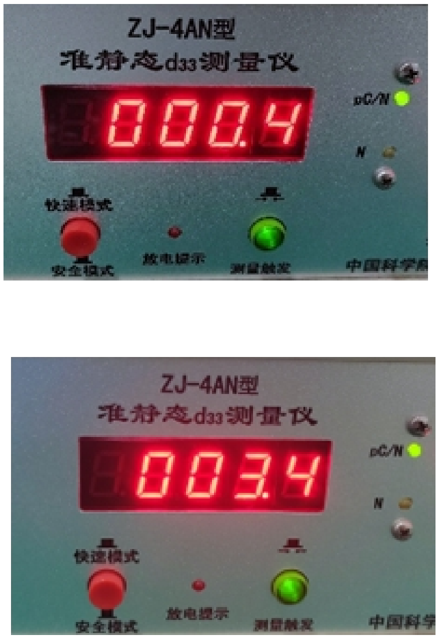

Fig. S15. The piezoelectric coefficient d_{33} value before polarization and after polarization with a 30 kV/cm electric field, respectively.

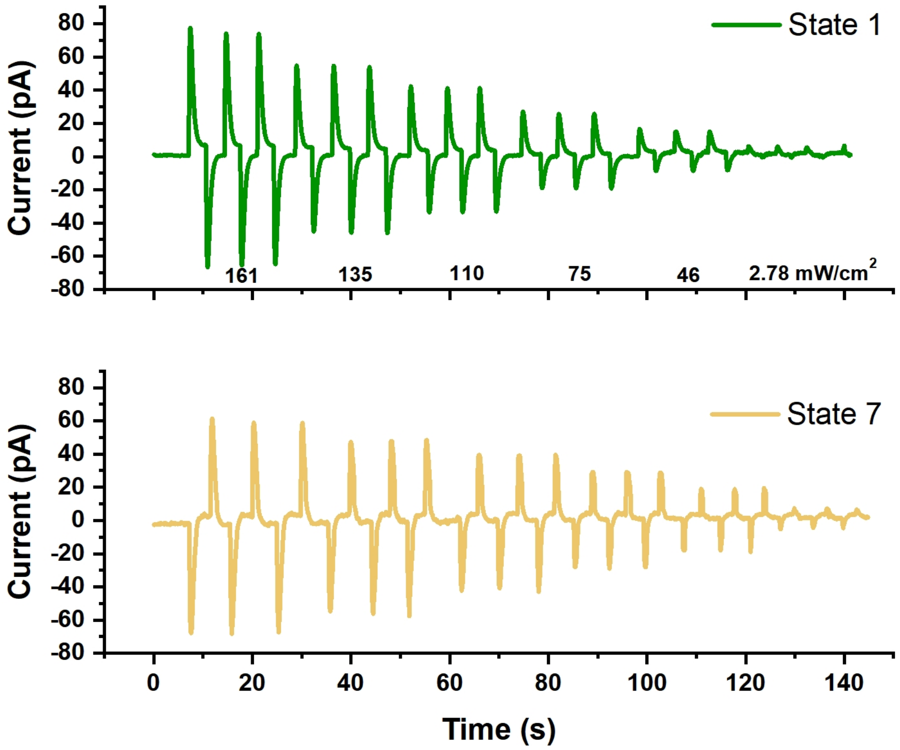

Fig. S16. Photo-pyroelectric currents measured under 404 nm laser illumination (State 1 and State 7).

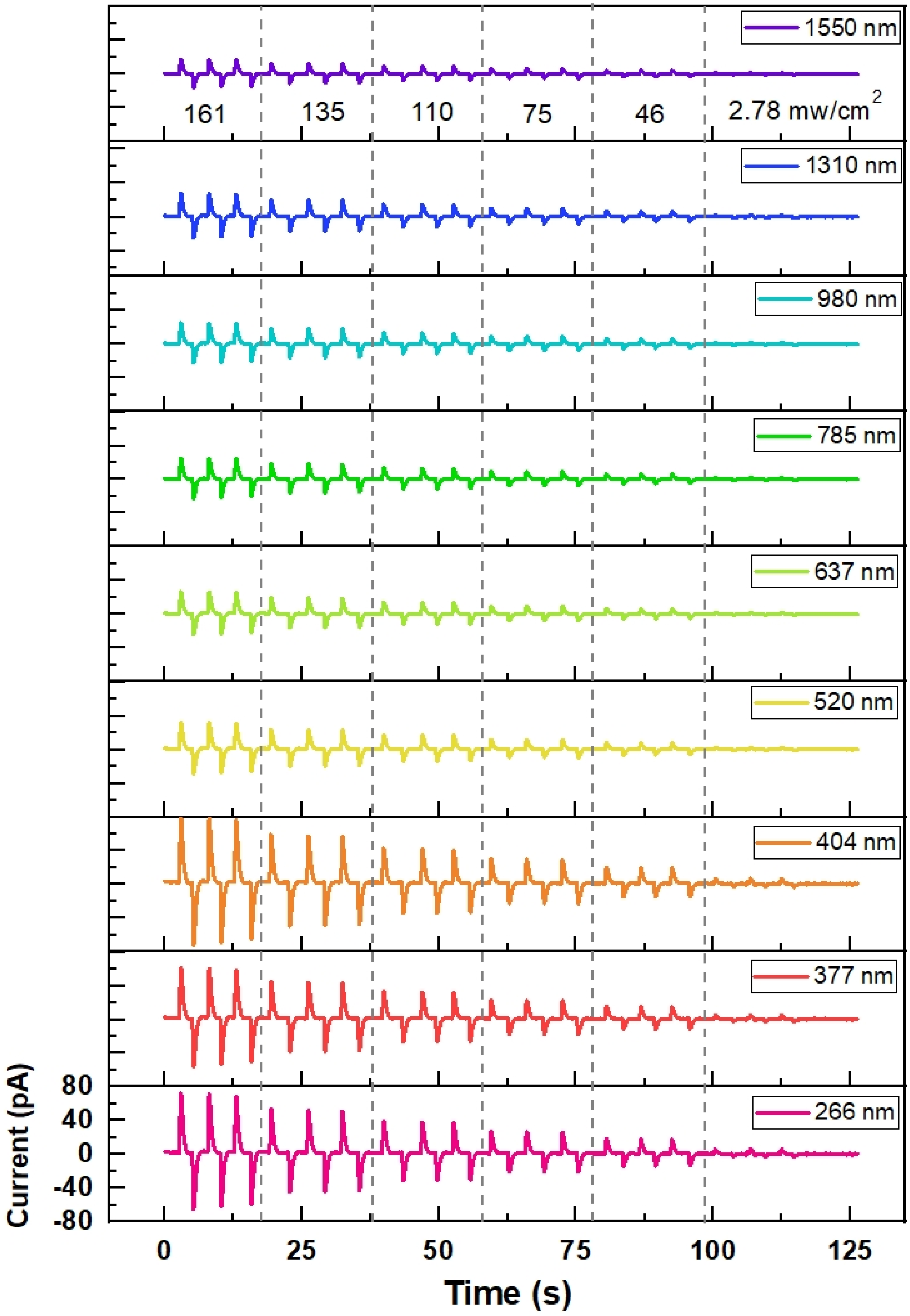

Fig. S17. Photo-pyroelectric currents measured under different wavelength.

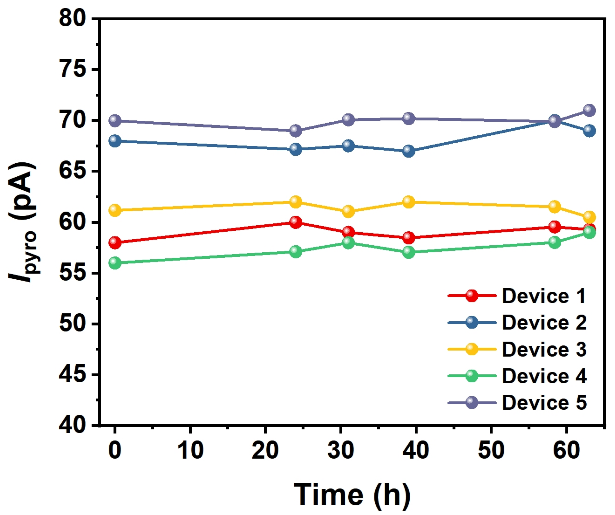

Fig. S18. Reproducible nature of the photo-pyroelectric currents generation in different devices (404 nm, 135 mW/cm^2).

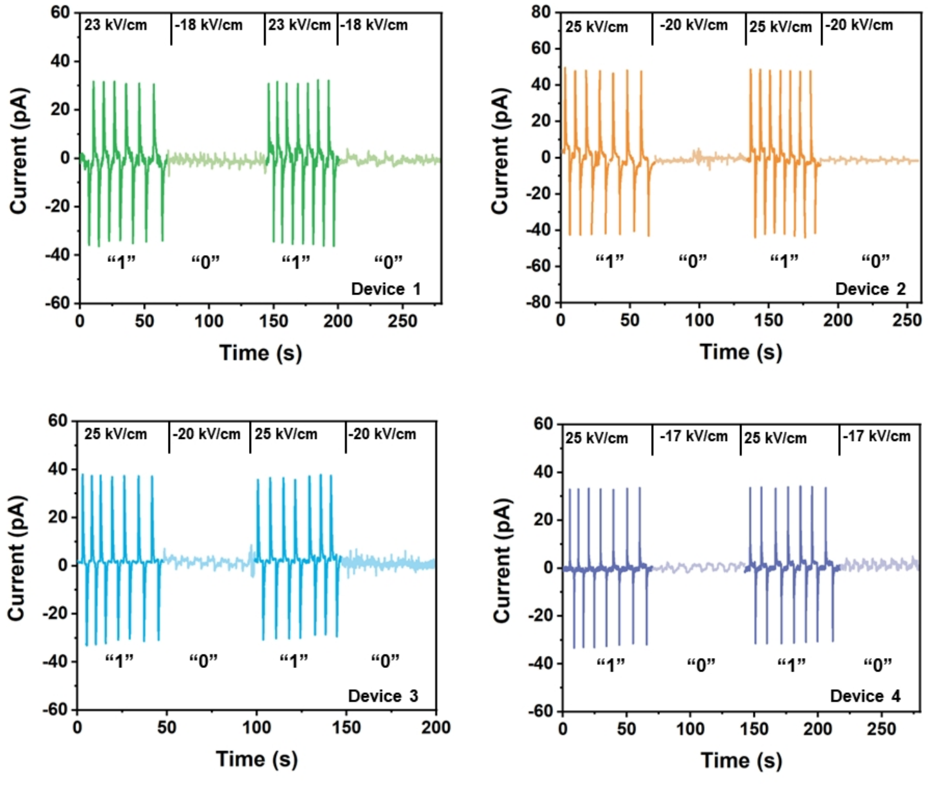

Fig. S19. Photo-pyroelectric currents of different devices were measured by polarization engineering (404 nm, 135 mW/cm^2).

Data S1. Other Supplementary Material for this manuscript contains the primary research data, including detailed ferroelectric property measurements and photo-pyroelectric response results.
